# Repair of partial atrioventricular septal defect through a modified right vertical infra-axillary thoracotomy: a single-center experience

**DOI:** 10.3389/fcvm.2026.1810648

**Published:** 2026-05-12

**Authors:** Heqi Zhang, Zehua Shao, Haoju Dong, Shubo Song, Fanwei Meng, Xiaoliang Qian, Taibing Fan, Weijie Liang

**Affiliations:** 1 Department of Children’s Heart Center, Central China Fuwai Hospital of Zhengzhou University, Zhengzhou, Henan, China; 2 Department of Surgery, Fuwai Hospital, National Center for Cardiovascular Diseases, Chinese Academy of Medical Sciences and Peking Union Medical College, Beijing, China; 3 Department of Structural Heart Center, Central China Fuwai Hospital of Zhengzhou University, Zhengzhou, Henan, China; 4 Department of Extracorporeal Circulation, Central China Fuwai Hospital of Zhengzhou University, Zhengzhou, Henan, China

**Keywords:** central cardiopulmonary bypass, congenital heart disease, minimally invasive cardiac surgery, modified right vertical infra-axillary thoracotomy, partial atrioventricular septal defect

## Abstract

**Background:**

The right vertical infra-axillary thoracotomy (RVIAT) approach has become an established surgical technique for common congenital heart diseases. In recent years, the traditional RVIAT approach has been refined by incorporating thoracoscopic concepts and techniques, resulting in the development of a modified RVIAT (MRVIAT) technique. This single-center observational study aimed to evaluate the clinical outcomes and surgical feasibility of MRVIAT for the repair of partial atrioventricular septal defect (PAVSD).

**Methods:**

A retrospective analysis was conducted to assess the perioperative outcomes of patients who underwent PAVSD repair using the MRVIAT technique. All procedures were completed through a single 2–5 cm incision under central cardiopulmonary bypass.

**Results:**

All 64 patients underwent successful repair without conversion to median sternotomy. No mortality or major complications were observed. The mean age was 12.8 ± 16.5 years (range, 0.3–63 years). The median body weight was 55.8 kg (range, 4.2–101.4 kg). The mean operative time was 177.0 ± 46.4 min. The mean cardiopulmonary bypass time was 98.2 ± 34.4 min, and the mean aortic cross-clamp time was 64.5 ± 23.1 min. One patient required reoperation due to patch dehiscence at the atrioventricular valve repair site. A sequential analysis of operative time demonstrated an overall downward trend with increasing case experience, suggesting the presence of a learning curve. During follow-up, no moderate or severe valvular regurgitation was detected, and cardiac function remained within normal limits in all patients.

**Conclusion:**

The MRVIAT technique appears to be a safe and effective approach for the repair of PAVSD across a wide age spectrum, from infancy to adulthood. The concealed incision offers favorable cosmetic outcomes and may improve patient satisfaction.

## 1 Introduction

Median sternotomy has long been the conventional approach for the correction of congenital heart defects (1). However, the resulting midline scar may be cosmetically undesirable and can cause psychological distress, particularly in young female patients (1). Minimally invasive cardiac surgery (MICS) refers to surgical procedures performed through smaller incisions and is associated with reduced surgical trauma compared with conventional median sternotomy (2). This approach has been shown to reduce hospital stay, the use of blood products, postoperative pain, and neurologic complications, thereby facilitating recovery and improving clinical outcomes (2–4). Several minimally invasive approaches have been adopted for the treatment of adult congenital heart disease, including the right infra-axillary incision, transverse intercostal incision, and totally thoracoscopic techniques (5–7). Although favorable results have been reported, certain technical limitations remain (5–7).

In recent years, the modified right vertical infra-axillary thoracotomy (MRVIAT) technique was developed by integrating the traditional right infra-axillary incision with thoracoscopic concepts. Surgical workflow was optimized, and modifications were made to surgical instruments, aortic occlusion strategies, and cardiopulmonary bypass cannulation techniques. These refinements expanded the surgical indications and enabled the repair of partial atrioventricular septal defect (PAVSD) across a broad age spectrum, from infancy to adulthood, through a single 2–5 cm incision under central cardiopulmonary bypass. Favorable clinical outcomes were achieved. This retrospective study aimed to evaluate the early clinical outcomes and surgical feasibility of MRVIAT in the treatment of adult PAVSD.

## 2 Methods

This study was approved by the Institutional Review Board of Central China Fuwai Hospital (Approval No. 2024-64). Written informed consent was obtained from all patients. Between January 2022 and December 2024, 64 consecutive patients with PAVSD underwent surgical repair using the MRVIAT technique at our institution. A learning-curve analysis was additionally performed using two complementary approaches: a scatter plot of operative time according to chronological case sequence with locally weighted scatterplot smoothing (LOWESS), and cumulative sum (CUSUM) analysis based on operative time. For CUSUM analysis, the deviation of each case-specific operative time from the overall mean operative time was sequentially accumulated, and the peak of the CUSUM curve was used to identify the transition point of the learning process.

Inclusion criteria were as follows: (1) a preoperative diagnosis of partial PAVSD, with or without additional septal defects; and (2) pulmonary hypertension with a pulmonary vascular resistance <5 Wood units confirmed by right heart catheterization. Exclusion criteria included: (1) complex congenital heart defects unsuitable for repair through the right infra-axillary approach, such as double outlet right ventricle; (2) severe pulmonary hypertension with right-to-left shunting; (3) thoracic deformities precluding right intercostal access; and (4) significant neurologic, respiratory, hepatic, or renal dysfunction.

In addition, all cases were required to be judged suitable for repair through the MRVIAT approach based on comprehensive preoperative imaging and overall anatomical assessment.

All patients underwent comprehensive preoperative evaluation, including physical examination, electrocardiography, chest radiography, transthoracic echocardiography, and cardiac computed tomography angiography. Baseline clinical characteristics are summarized in Table 1.

**Table 1:** Preoperative general baseline data of patients.

Indicator	(*n* = 64)
Gender (Male, *n* %)	21 (32.8%)
Weight, kg	55.8 (49.5, 65.1)
Age, year	12.8 ± 16.5
<18 years	22 (34.4%)
≥18 years	42 (65.6%)
Preoperative cardiac catheterization, *n* (%)	1 (1.6%)
Size of the primary atrial septal defect, *n* (%)	21.0 (18.1, 28.8)
Preoperative LVEF, %	67.1 ± 3.5
Preoperative congenital anomalies, *n* (%)	
Secundum atrial septal defect	8 (12.5%)
Patent foramen ovale	12 (18.8%)
Preoperative Pulmonary Arterial Hypertension, *n* (%)	
Mild	14 (21.9%)
Moderate	16 (25.0%)
Severe	1 (4.5%)
Preoperative Mitral Valve Regurgitation, *n* (%)	
Moderate	46 (71.9%)
Severe	15 (23.4%)
Preoperative Tricuspid Valve Regurgitation, *n* (%)	
Moderate	50 (78.1%)
Severe	11(17.2%)

### 2.1 Procedure

After induction of combined intravenous and inhalational anesthesia, endotracheal intubation was performed. Invasive arterial monitoring was established via the left radial artery, and a central venous catheter was inserted into the right internal jugular vein. A nasopharyngeal temperature probe was placed for continuous temperature monitoring, and a urinary catheter was inserted.

**Figure 1:**
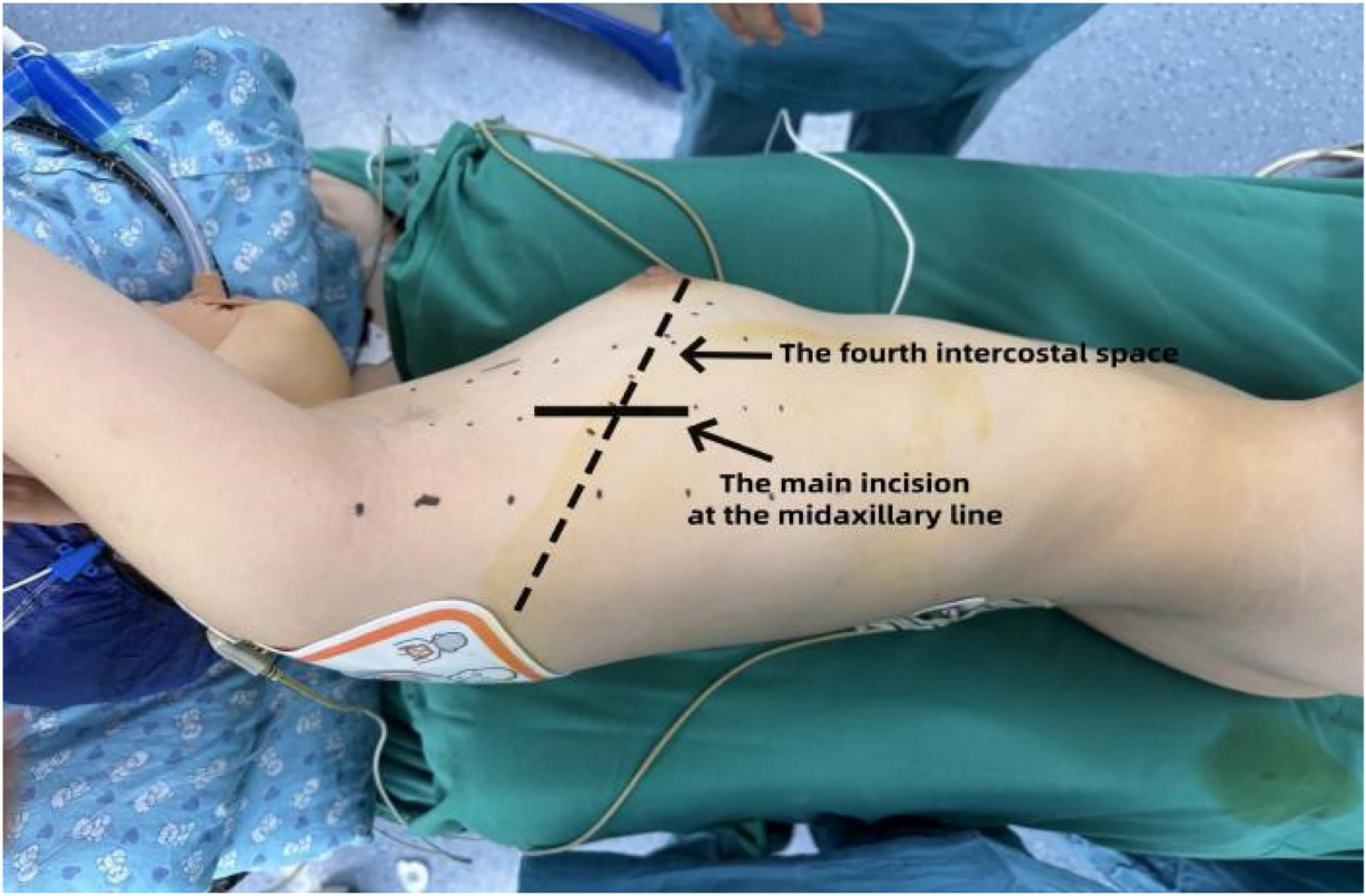
Preoperative patient positioning.

The patient was positioned in the left lateral decubitus position, with the right arm abducted and the elbow flexed and secured to a head frame to expose the axillary region (Figure 1). After standard skin preparation and draping, the right arm was released and allowed to rest naturally to avoid brachial plexus injury.

**Figure 2:**
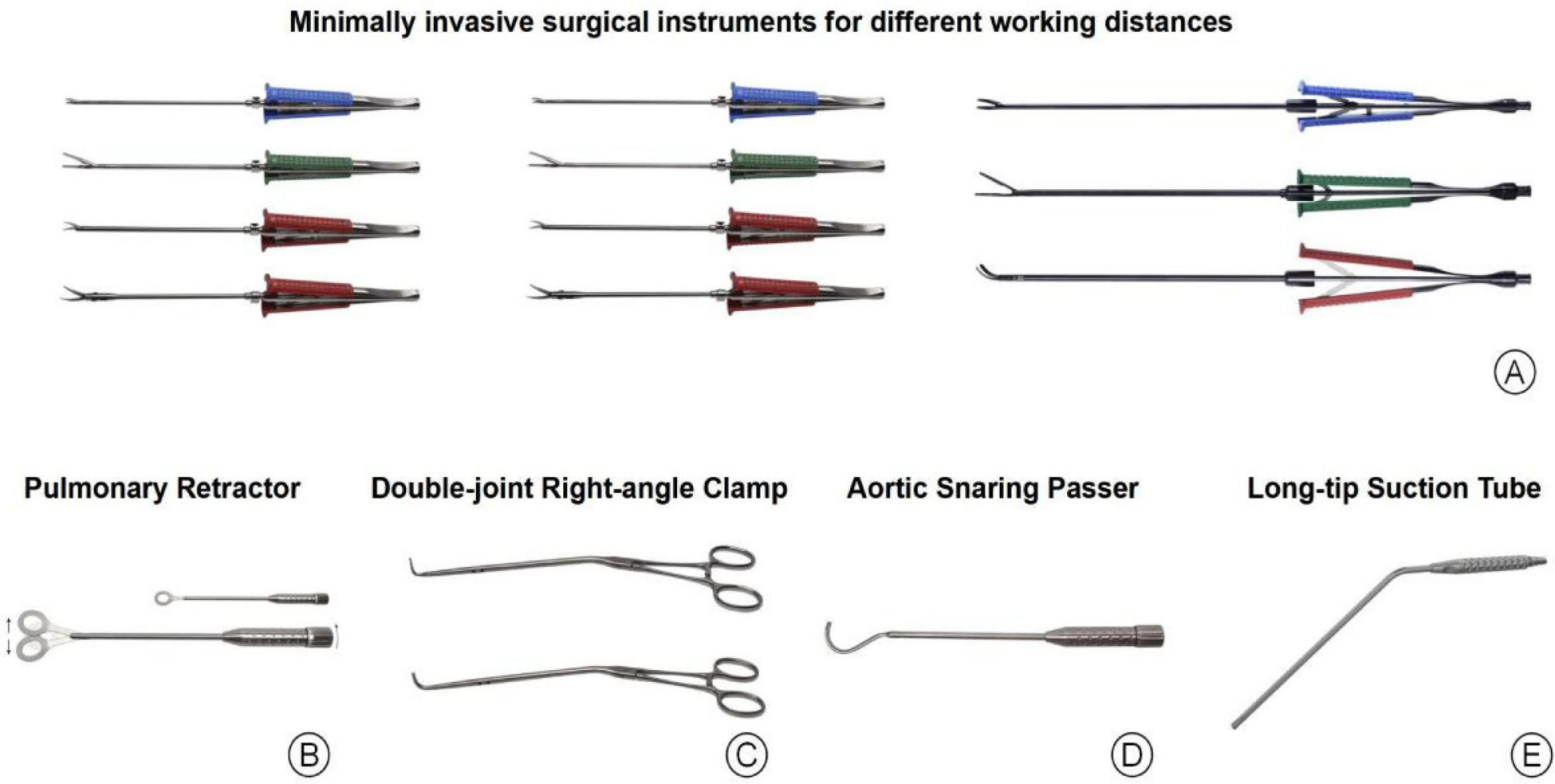
Specialized minimally invasive surgical instruments. **(A)** Long-shaft minimally invasive instruments designed for different age groups. **(B)** Lung retractor used to protect the right lung. **(C)** Double-joint right-angle clamp. **(D)** Aortic occlusion band passer. **(E)** Extended suction tip for deep operative field exposure.

A 2–5 cm longitudinal incision was made along the midaxillary line in the fourth intercostal space. The subcutaneous tissue was dissected, and partial division of the serratus anterior and intercostal muscles was performed. After temporary cessation of ventilation, the thoracic cavity was entered through the fourth intercostal space. A rib retractor was applied, and a wound protector was inserted. The right lung was gently retracted and protected using a lung retractor and moist gauze (Figure 2).

The pericardium was opened longitudinally approximately 2 cm anterior to the right phrenic nerve and suspended with traction sutures. Continuous carbon dioxide insufflation was administered into the thoracic cavity to reduce the risk of air embolism. The ascending aorta and main pulmonary artery were fully mobilized. An aortic occlusion band was passed between the aorta and pulmonary artery and secured with a tourniquet.

**Figure 3:**
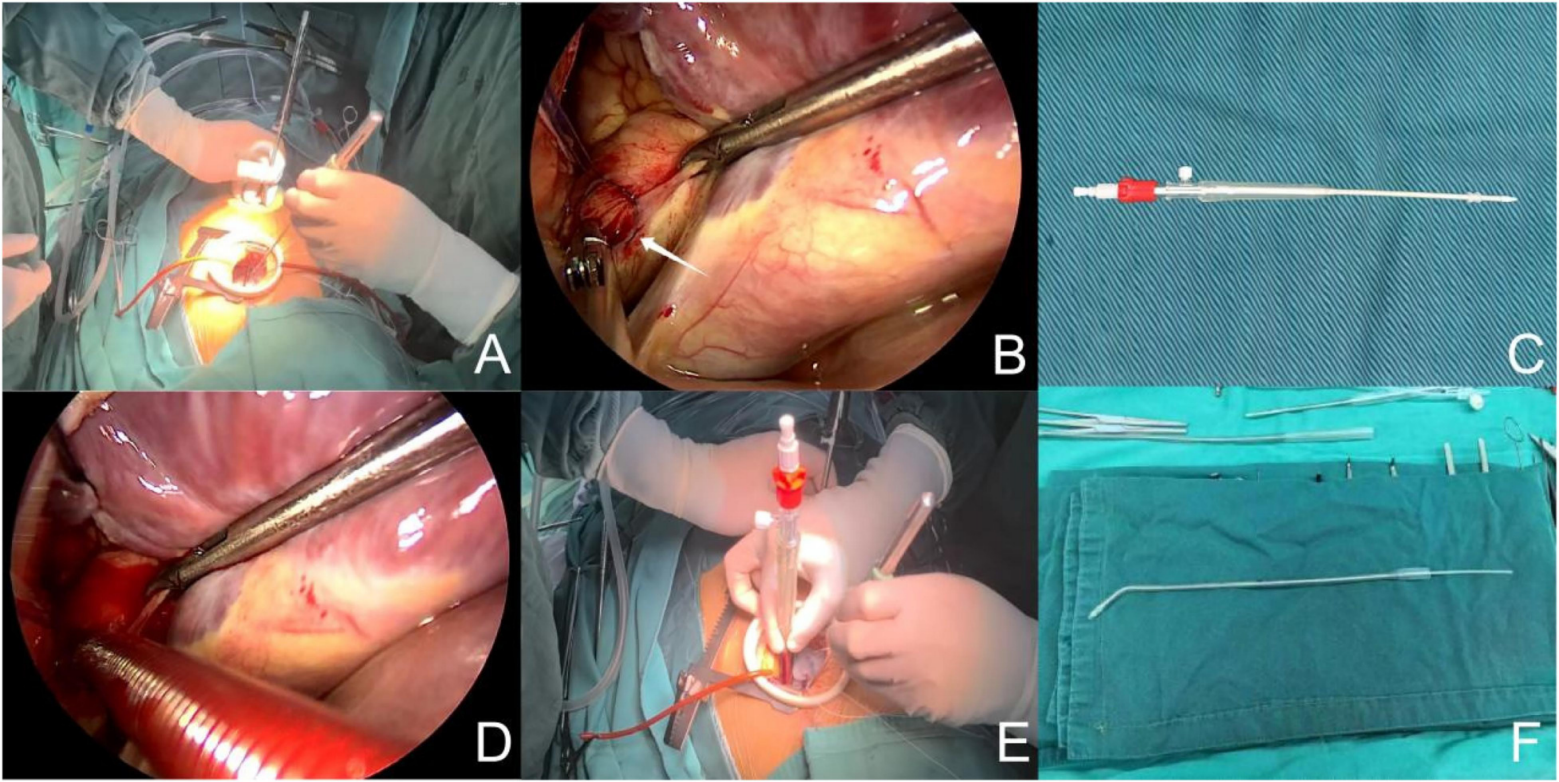
Aortic exposure and cannulation through the drainage port. **(A)** The assistant retracting the long-shaft needle holder. **(B)** The needle holder lifting the adventitia of the ascending aorta. **(C)** A 19-Fr arterial cannula. **(D,E)** Placement of purse-string sutures and aortic cannulation performed by the primary surgeon. **(F)** Adjustable curved inferior vena cava cannula.

In adult or larger patients (usually >50 kg), a long-shaft minimally invasive needle holder was introduced through the drainage port. The adventitia of the ascending aorta was grasped and gently retracted inferiorly by the first assistant to expose the aortic cannulation site (Figures 3A–E). Double purse-string sutures were placed, and aortic cannulation was performed (a 19-Fr arterial cannula was typically used in adults). The superior and inferior venae cavae were then cannulated separately. A metal right-angle superior vena cava cannula was generally selected for the superior vena cava, and an adjustable curved inferior vena cava cannula with an internal stylet was used for the inferior vena cava (Figure 3F). Both venous cannulas were exteriorized through the drainage port.

**Figure 4:**
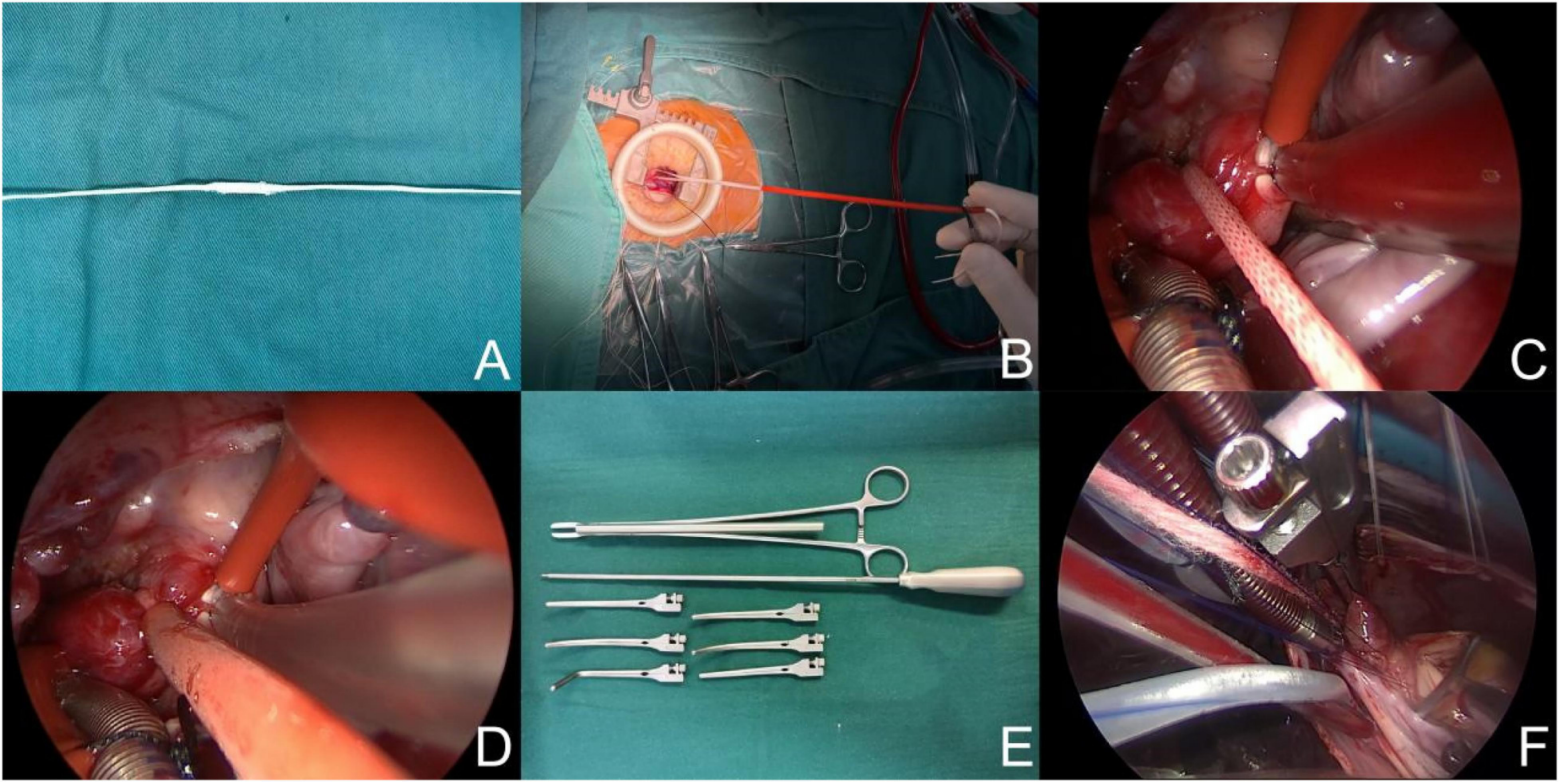
Aortic occlusion techniques. **(A)** Aortic occlusion band. **(B–D)** Sequential steps of ascending aortic occlusion using the occlusion band. **(E,F)** Detachable aortic cross-clamp.

After initiation of cardiopulmonary bypass, the superior and inferior venae cavae were snared to achieve total venous occlusion. A purse-string suture was placed at the aortic root for insertion of the cardioplegia needle. After reduction of flow, the ascending aorta was occluded using either the occlusion band or a detachable aortic cross-clamp (Figure 4). Cardioplegic solution was administered via the aortic root to achieve cardiac arrest.

A right atriotomy was performed. The cleft of the anterior mitral leaflet was repaired with five interrupted sutures reinforced with autologous pericardial pledgets. Tricuspid annuloplasty was performed using interrupted mattress sutures placed at the posterior annulus and commissural region. A saline test confirmed adequate valve competence. A bovine pericardial patch of appropriate size was then used to repair the primum atrial septal defect with continuous suturing along the septal leaflet margin. In one patient with posterior leaflet hypoplasia identified preoperatively by transthoracic echocardiography, autologous pericardial augmentation of the posterior leaflet was performed, and the saline test confirmed satisfactory valve competence.

The cleft was closed with interrupted sutures and reinforced with autologous pericardial pledgets. Saline testing and intraoperative transesophageal echocardiography were used to assess leaflet coaptation and to avoid overcorrection or left atrioventricular valve stenosis.

De-airing was performed via the left ventricular vent and the aortic root. The foramen ovale was closed with continuous suturing (Supplementary Video S1). After complete de-airing, cardiopulmonary bypass was discontinued. Following release of the aortic occlusion, spontaneous return of sinus rhythm was observed. Modified ultrafiltration was performed before complete separation from cardiopulmonary bypass.

**Figure 5:**
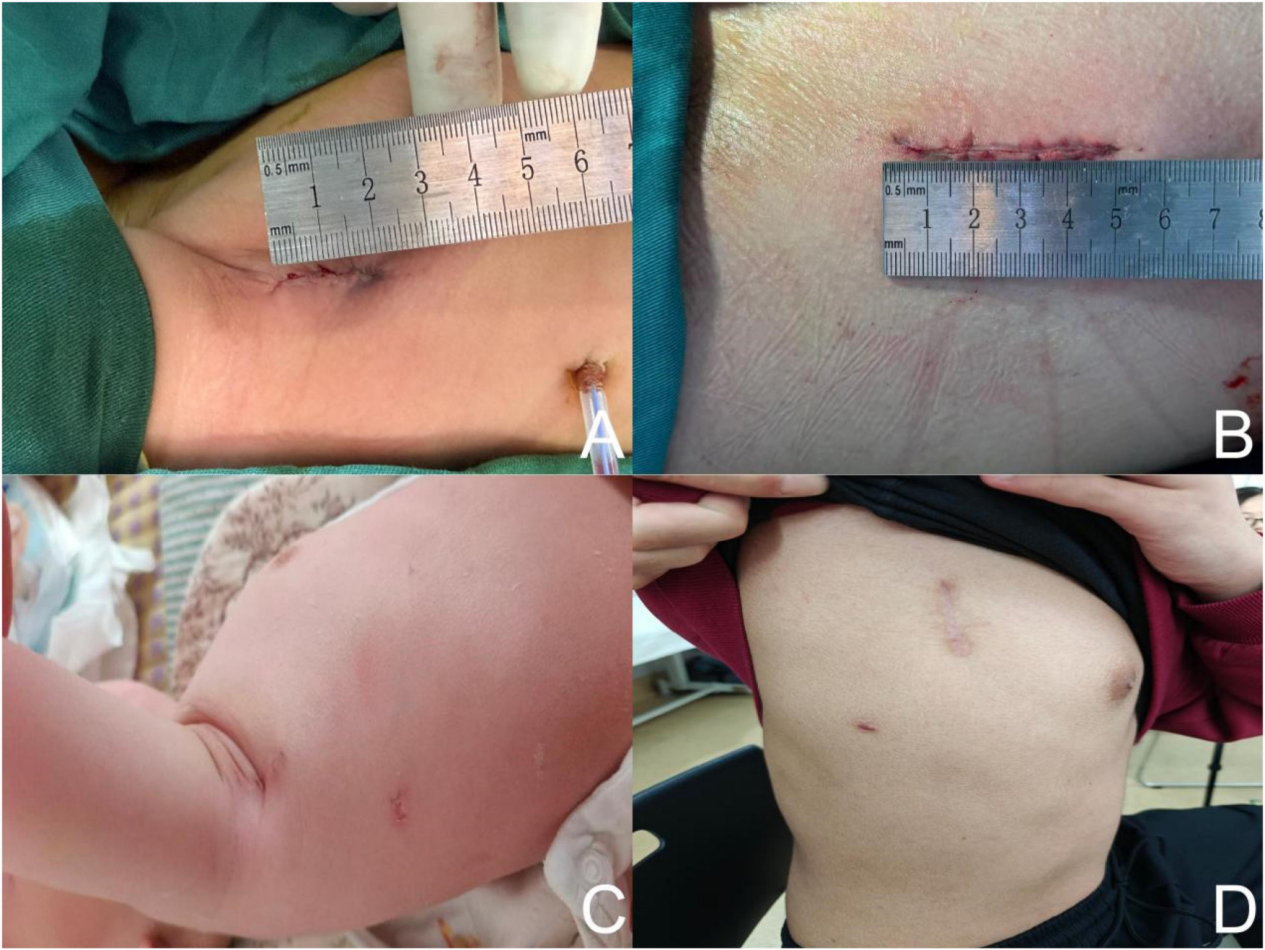
Postoperative incision appearance. **(A,B)** Postoperative incision in a 1-year-old patient, with an approximate length of 2.5 cm. **(C,D)** Postoperative incision in an 18-year-old female patient (80 kg), measuring approximately 5 cm. Photographs were obtained 3 months after surgery. The incision is located along the midaxillary line and is concealed by the naturally resting arm.

Intraoperative transesophageal echocardiography confirmed the absence of residual shunt or significant valvular regurgitation prior to weaning from cardiopulmonary bypass. After meticulous hemostasis, a right pleural drainage tube was placed through the same incision. The lungs were fully re-expanded, and the intercostal space was closed with sutures. The subcutaneous tissue and skin were closed using absorbable sutures (Figure 5). The patient was subsequently transferred to the intensive care unit for postoperative management.

During weaning from cardiopulmonary bypass, adequacy of left atrioventricular valve repair was judged by integrating intraoperative transesophageal echocardiography with hemodynamic findings. Trivial or mild residual regurgitation with satisfactory leaflet coaptation, no significant transvalvular stenosis, and stable hemodynamics under low-dose vasoactive support was considered acceptable. In contrast, moderate or greater residual regurgitation, a marked eccentric jet suggesting inadequate repair, significant stenosis, difficulty separating from bypass, or persistent hemodynamic instability requiring substantial vasoactive support prompted consideration of a second run of cardiopulmonary bypass for further revision.

Perioperative pulmonary hypertension management was individualized. In most infants and young children in this cohort, elevated pulmonary artery pressure was considered secondary to left-to-right shunting and/or left atrioventricular valve regurgitation rather than fixed pulmonary vascular disease. Therefore, pulmonary vasodilators were not routinely administered preoperatively or postoperatively. Management focused on correction of the underlying lesion, optimized ventilation, adequate sedation and analgesia, maintenance of acid-base balance, and avoidance of hypoxemia, hypercapnia, and pulmonary vasoconstriction. Postoperative bedside echocardiography was routinely used to reassess pulmonary artery pressure and residual valve regurgitation; when pulmonary pressure was not significantly elevated and hemodynamics were stable, no routine pulmonary vasodilator therapy was required. Targeted pulmonary vasodilator treatment was reserved for selected patients with persistently elevated pulmonary artery pressure or hemodynamic concern.

### 2.2 Major technical modifications

#### 2.2.1 Modified surgical instruments

dedicated minimally invasive instruments were developed through interdisciplinary collaboration to accommodate the right infra-axillary approach across different age groups. Long-shaft instruments with varying working lengths were designed to address differences in operative depth (Figure 2A). Additional devices, including a lung retractor, an aortic band passer, and a double-joint right-angle clamp, were introduced to improve exposure and instrument maneuverability (Figures 2B–E). These modifications enhanced operative precision and facilitated intracardiac repair through a limited 2–5 cm incision.

#### 2.2.3 Modified aortic occlusion technique

In patients weighing ≤30 kg, ascending aortic occlusion was typically achieved using a braided polyester occlusion band. Following pericardial suspension, the ascending aorta and pulmonary artery were mobilized, and the occlusion band was secured prior to cardioplegic infusion (Figures 2C,D). In patients weighing >30 kg, a detachable aortic cross-clamp or a double-band occlusion technique was selected according to operative requirements. This strategy improved visualization within the restricted operative field and streamlined procedural steps.

The approximate 30-kg threshold was based on our institutional experience. In patients weighing <30 kg, the smaller ascending aortic diameter and relatively shallower operative depth generally make braided polyester band occlusion more practical and reliable. In heavier patients, a detachable cross-clamp or double-band strategy was selected according to exposure and operative convenience to ensure dependable myocardial protection and procedural efficiency.

#### 2.2.3 Modified cardiopulmonary bypass cannulation

Arterial and venous cannulae one size smaller than conventional were selected based on peripheral cardiopulmonary bypass principles. Vacuum-assisted venous drainage (VAVD) was routinely applied to maintain adequate venous return. In adult patients, a 19-Fr arterial cannula and 20-Fr superior and inferior vena cava cannulae were sufficient to achieve target flow rates when combined with VAVD. A blunt-tip arterial cannula was preferred to reduce the risk of aortic dissection or posterior wall injury during cannulation.

#### 2.2.4 Application of thoracoscopic assistance

Conventional adult cardiac surgical instruments are primarily designed for median sternotomy, where exposure is wide and operative distance is short. In contrast, the right infra-axillary approach involves a deeper operative field and extended working distance. Thoracoscopic instruments provide improved access to deep intracardiac structures. Placement of a thoracoscopic camera through the drainage port provided stable illumination and enhanced visualization, thereby overcoming exposure limitations inherent to the limited incision.

In our experience, establishment of cardiopulmonary bypass was still performed predominantly under direct vision, including aortic and bicaval cannulation. Thoracoscopic assistance was not routinely required in all patients, but was mainly used in larger patients, in whom the deeper thoracic cavity and longer working distance could limit exposure with conventional RVIAT. Under these circumstances, thoracoscopic assistance improved deep illumination and visualization of intracardiac structures and was mainly used to facilitate intracardiac repair, whereas the principal repair was still completed through the working incision.

## 3 Results

### 3.1 Baseline characteristics

The mean age was 12.8 ± 16.5 years, and the median body weight was 55.8 kg (range, 4.2–101.4 kg). The mean preoperative left ventricular ejection fraction (LVEF) was 67.1 ± 3.5%. The median diameter of the primum atrial septal defect was 21.0 mm (interquartile range, 18.1–28.8 mm). Concomitant secundum atrial septal defect was present in 8 patients (12.5%), and patent foramen ovale in 12 patients (18.8%). Moderate mitral regurgitation was observed in 46 patients (71.9%), and severe mitral regurgitation in 15 patients (23.4%). Moderate tricuspid regurgitation was present in 50 patients (78.1%), and severe tricuspid regurgitation in 11 patients (17.2%). Mild pulmonary hypertension was observed in 14 patients (21.9%), moderate in 16 patients (25.0%), and severe in 1 patient (1.6%). The patient with severe pulmonary hypertension underwent right heart catheterization, which demonstrated a pulmonary vascular resistance of 1.4 Wood units after oxygen inhalation, and subsequently proceeded to surgical repair. Among the 15 patients with severe left atrioventricular valve regurgitation, the predominant mechanism was anterior leaflet cleft; one patient additionally had posterior leaflet hypoplasia that had been recognized preoperatively.

#### 3.1.1 Perioperative outcomes and postoperative complications

**Figure 6:**
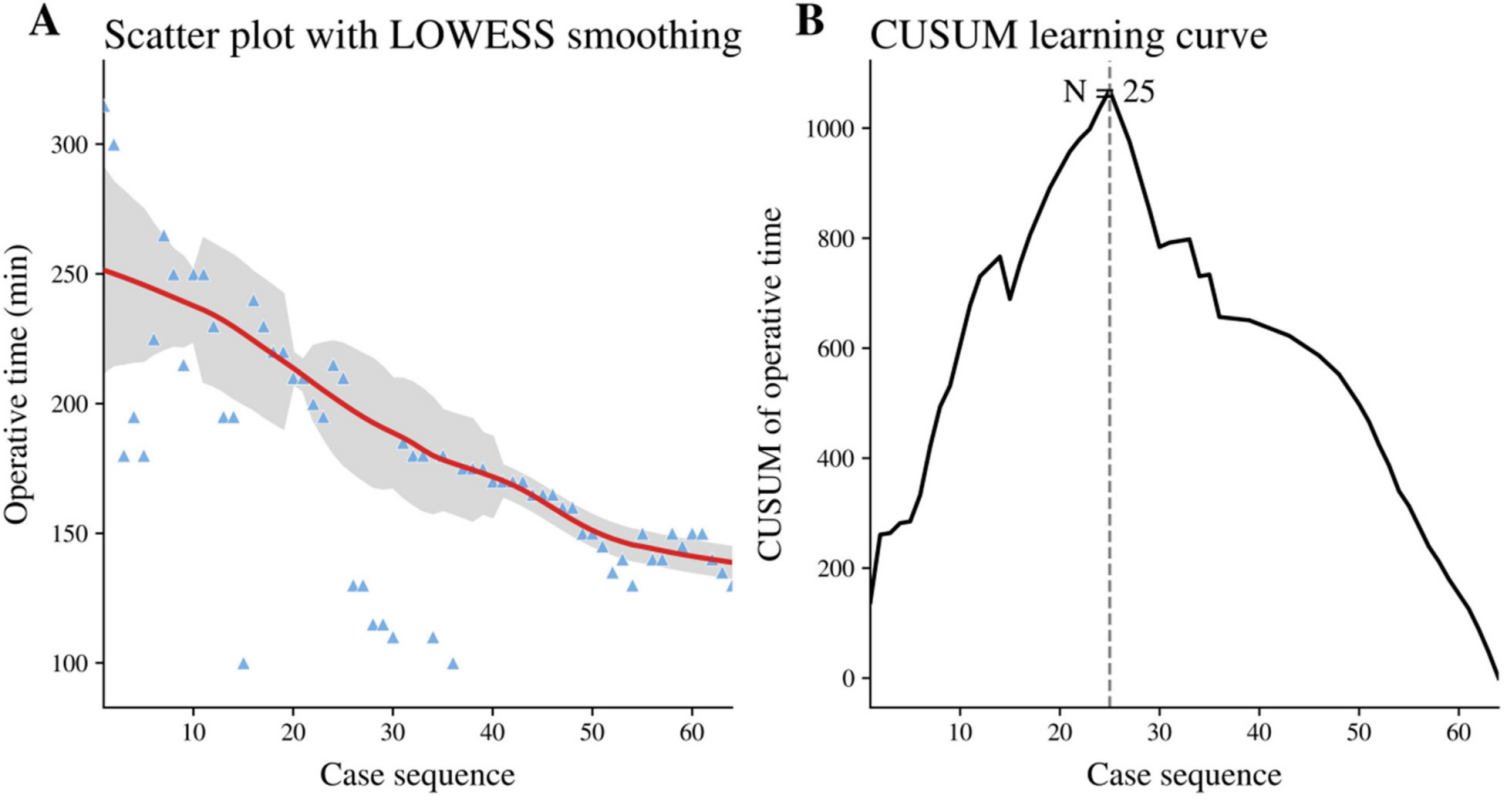
Learning-curve analysis of operative time in sequential PAVSD repairs performed using MRVIAT. **(A)** Scatter plot of operative time versus case sequence with LOWESS smoothing. **(B)** CUSUM learning curve based on operative time, showing a transition point at case 25.

All 64 patients successfully underwent the planned procedure without conversion to median sternotomy. All perioperative outcomes and postoperative complications are summarized in Table 2. The mean operative time was 177.0 ± 46.4 min. The mean cardiopulmonary bypass time was 98.2 ± 34.4 min, and the mean aortic cross-clamp time was 64.5 ± 23.1 min. The mean chest drainage volume within the first 24 h was 140.6 ± 121.4 mL. The mean duration of postoperative mechanical ventilation was 7.8 ± 5.9 h, and the mean intensive care unit stay was 32.9 ± 12.1 h. The mean postoperative incision length was 3.7 ± 1.2 cm, and the mean postoperative hospital stay was 8.2 ± 2.5 days. No patient developed definite postoperative pneumonia or other severe pulmonary infectious complications based on clinical documentation. LOWESS smoothing demonstrated an overall reduction in operative time with increasing case experience. Consistently, the CUSUM curve reached its maximum at case 25, suggesting a transition from the initial learning phase to a more stable and proficient stage thereafter (Figure 6).

**Table 2:** Perioperative and postoperative complications, follow-up results of patients.

Indicator	(*n* = 64)
Operation time, min	177.0 ± 46.4
Cardiopulmonary bypass time, min	98.2 ± 34.4
Aortic cross-clamp time, min	64.5 ± 23.1
Postoperative chest drainage volume within 24-hour, ml	140.6 ± 121.4
Postoperative mechanical ventilation time, h	7.8 ± 5.9
Postoperative ICU stay Time, h	32.9 ± 12.1
Postoperative incision length, cm	3.7 ± 1.2
Postoperative hospital stay time, d	8.2 ± 2.5
Postoperative LVEF,%	68.5 ± 3.5
Patient/family satisfaction/Yes, *n* (%)	64 (100%)
Reoperation, *n* (%)	1 (1.6%)
Postoperative Complications	
Moderate or Severe Mitral Valve Regurgitation, *n* (%)	1 (1.6%)
Moderate or Severe Tricuspid Valve Regurgitation, *n* (%)	1 (1.6%)
Residual Flow, *n* (%)	0
Cardiac Arrhythmia, *n* (%)	0
Low Cardiac Output Syndrome, *n* (%)	0
Neurological Disorders, *n* (%)	0
Dead, *n* (%)	0
Permanent atrioventricular block, *n* (%)	0
Phrenic nerve injury, *n* (%)	0
Incision infection, *n* (%)	0
Winged scapula, *n* (%)	0
Poor wound healing, *n* (%)	0

One patient developed severe valvular regurgitation on postoperative day 2, likely due to suture dehiscence, and underwent emergency valve repair through the original incision. No mortality or major complications were observed during hospitalization or follow-up. No significant residual shunt (>2 mm by transthoracic echocardiography), heart failure, or severe arrhythmia was documented. No permanent atrioventricular block, phrenic nerve injury, incision infection, winged scapula, or poor wound healing was observed in this cohort. Perioperative and postoperative complications, follow-up results, and patient/family satisfaction are summarized in Table 2.

#### 3.1.2 Postoperative follow-up

Follow-up data were available for 63 patients (98.4%). The median follow-up duration was 1.3 years (range, 0.3–2.5 years). No surgery-related mortality, progression of valvular regurgitation, or reintervention was observed during follow-up.

One patient developed mild-to-moderate mitral regurgitation following mitral valve repair. The degree of regurgitation remained stable throughout the follow-up period and is currently under continued observation. No residual shunt was detected on follow-up echocardiography. In addition, no incision-related complications or wound healing problems were documented.

## 4 Discussion

Median sternotomy provides excellent surgical exposure and remains the standard approach for most cardiac procedures. However, it is associated with greater surgical trauma, increased bleeding, prolonged hospitalization, and visible scarring (5, 8). Over the past two decades, various minimally invasive approaches have been introduced in congenital heart surgery, including lower partial sternotomy, subxiphoid access, anterior or lateral thoracotomy, and axillary incision techniques (9–12). The primary objectives of these techniques are to improve cosmetic outcomes, reduce postoperative pain, and shorten recovery and hospital stay. Among these benefits, improved cosmetic appearance has consistently been reported as the most evident advantage (1).

The right vertical infra-axillary thoracotomy approach was initially applied in pediatric patients for atrial septal defect repair. With increasing experience, its indications were expanded to include valve repair, tetralogy of Fallot, and partial anomalous pulmonary venous connection (13). In adult patients, exposure is more challenging due to increased thoracic depth and limited rib elasticity. Previously, a body weight >30 kg was considered a relative contraindication (5). However, accumulated experience suggests that higher body weight is not an absolute limitation, as demonstrated in the present series. Although no patient in the present cohort had persistent left superior vena cava, our prior experience with other RVIAT procedures suggests that this anatomic variation can be managed through coronary sinus venting or temporary control of the left superior vena cava after adequate mobilization when necessary.

Totally thoracoscopic cardiac surgery can be performed through three small incisions measuring approximately 1.0–2.5 cm (14). Although this technique minimizes surgical trauma and postoperative pain (15), the absence of depth perception increases the learning curve and may prolong operative time (16).

Peripheral cannulation is widely adopted in adult congenital cardiac surgery (16, 17). Nevertheless, risks such as retrograde aortic dissection, embolism, and limb ischemia remain. Stroke incidence associated with femoral cannulation has been reported at approximately 1.17% (2, 18). By utilizing central cannulation, the MRVIAT approach may reduce complications related to peripheral access while avoiding groin scarring.

At our center, we generally favor a totally central cannulation strategy in order to preserve the single-incision, single-field concept, avoid an additional groin incision and scar, and minimize potential complications related to femoral venous cannulation, such as vascular injury and lower-extremity morbidity. In the present study, all patients underwent central cannulation, and routine conversion to peripheral venous cannulation was not required because of inadequate venous drainage or limited exposure. Nevertheless, peripheral venous cannulation combined with central arterial cannulation remains a reasonable alternative in selected larger patients, depending on patient anatomy and institutional experience.

The MRVIAT technique integrates the right infra-axillary approach with thoracoscopic assistance. Through technical refinements, intracardiac repair can be completed through a single 2–5 cm incision. Compared with totally thoracoscopic techniques, this approach has been associated with shorter operative, cardiopulmonary bypass, and aortic cross-clamp times (19). In addition, soft tissue disruption is minimized, and favorable cosmetic outcomes are achieved. In addition, the learning-curve analysis showed a progressive reduction in operative time, and the CUSUM curve identified a transition point at approximately the 25th case, supporting the reproducibility of this approach after an initial learning phase. Although the mean cardiopulmonary bypass time in our cohort was 98.2 ± 34.4 min, careful attention should still be paid to pulmonary management, because prolonged cardiopulmonary bypass has been associated with an increased risk of postoperative pneumonia. Kilic et al. reported that cardiopulmonary bypass duration exceeding 100 min was associated with a higher risk of postoperative pneumonia after cardiac surgery (20). In the present series, no definite postoperative pneumonia was identified; however, transient increases in inflammatory markers or airway secretions were occasionally observed and improved with routine respiratory care, nebulization, and, when indicated, empiric anti-infective treatment. These findings underscore the importance of minimizing bypass duration and strengthening perioperative pulmonary surveillance, particularly in technically demanding cases.

Body weight influenced our technical strategy, particularly the selection of cannulation and aortic occlusion methods. However, because of the limited sample size and the small number of adverse events, formal weight-stratified comparative analysis was not performed in this study. Accordingly, the present findings should be interpreted as evidence supporting feasibility and safety in selected patients rather than superiority over other minimally invasive approaches.

Overall, MRVIAT without peripheral cannulation demonstrated satisfactory early clinical outcomes in the present cohort, with no operative mortality or major complications. This technique may represent a feasible alternative to conventional median sternotomy in selected patients with PAVSD. Previous studies have also demonstrated the feasibility and safety of minimally invasive approaches for the repair of congenital heart defects and atrioventricular septal defects, supporting the continued development of less invasive surgical strategies in appropriately selected patients (21–25).

## 5 Conclusions

The MRVIAT technique appears to be a safe and effective approach for the repair of PAVSD. The concealed incision provides favorable cosmetic outcomes and may contribute to improved patient satisfaction. With increasing experience, operative efficiency improved over time, supporting the feasibility of broader adoption in appropriately selected patients.

### Limitations

This study is limited by its single-center, retrospective design. The relatively small sample size (*n* = 64) may limit the generalizability of the findings. Larger prospective, multicenter studies are warranted to validate the reproducibility and external applicability of these results. In addition, longer follow-up is required to fully evaluate the durability and long-term outcomes of the MRVIAT approach. In addition, no formal subgroup analysis according to body weight was performed, which limits a more detailed assessment of how patient size may influence exposure, cannulation strategy, and outcomes.

## Data Availability

The raw data supporting the conclusions of this article will be made available by the authors, without undue reservation.
